# Supplementary material for: Randomised controlled trial of automated VR therapy to improve positive self-beliefs and psychological well-being in young people diagnosed with psychosis: a study protocol for the Phoenix VR self-confidence therapy trial
Source: BMJ Open. 2023 Dec 7;13(12):e076559. doi: 10.1136/bmjopen-2023-076559 (PMC10711910; doi:10.1136/bmjopen-2023-076559)
Supplement: Author's manuscript [file bmjopen-2023-076559.draft_revisions.pdf]

BMJ Open

BMJ Open is committed to open peer review. As part of this commitment we make the peer review history of every article we publish publicly available.

When an article is published we post the peer reviewers' comments and the authors' responses online. We also post the versions of the paper that were used during peer review. These are the versions that the peer review comments apply to.

The versions of the paper that follow are the versions that were submitted during the peer review process. They are not the versions of record or the final published versions. They should not be cited or distributed as the published version of this manuscript.

BMJ Open is an open access journal and the full, final, typeset and author-corrected version of record of the manuscript is available on our site with no access controls, subscription charges or pay-per-view fees (http://bmjopen.bmj.com).

If you have any questions on BMJ Open's open peer review process please email info.bmjopen@bmj.com

BMJ Open

A Randomised Controlled Trial of Automated VR Therapy to Improve Positive Self-Beliefs and Psychological Wellbeing in Young People Diagnosed with Psychosis: Study Protocol for the Phoenix VR Self-Confidence Therapy Trial

Journal:	*BMJ Open*
Manuscript ID	bmjopen-2023-076559
Article Type:	Protocol
Date Submitted by the Author:	10-Jun-2023

Complete List of Authors:	Freeman, Daniel; University of Oxford, Department of Psychiatry<br>Freeman, Jason; University of Oxford, Experimental Psychology<br>Rovira, Aitor; University of Oxford, Department of Experimental Psychology<br>Miguel, Andre Lages; University of Oxford, Department of Experimental Psychology<br>Ward, Rupert; University of Oxford, Department of Experimental Psychology<br>Bousfield, Matthew; University of Oxford, Department of Experimental Psychology<br>Riffiod, Ludovic; University of Oxford, Department of Experimental Psychology<br>Leal, Jose; University of Oxford, Nuffield Department of Population Health<br>Kabir, Thomas; The McPin Foundation<br>Yu, Ly-Mee; University of Oxford, Department of Primary Care Health Sciences<br>Waite, Felicity; University of Oxford Department of Psychiatry,<br>Rosebrock, Laina; University of Oxford, Department of Experimental Psychology

Keywords:	Schizophrenia & psychotic disorders < PSYCHIATRY, Virtual Reality, Clinical Trial

SCHOLARONE™
Manuscripts

A Randomised Controlled Trial of Automated VR Therapy to Improve Positive Self-Beliefs and Psychological Wellbeing in Young People Diagnosed with Psychosis: Study Protocol for the Phoenix VR Self-Confidence Therapy Trial

Daniel Freeman[1,2]*, Jason Freeman[1], Aitor Rovira[1,2], Andre Lages Miguel[1], Rupert Ward[1,2], Matthew Bousfield[1], Ludovic Riffiod[1], José Leal[3], Thomas Kabir[4], Ly-Mee Yu[5], Felicity Waite[1,2], Laina Rosebrock[1,2]

For peer review only

SUBMITTED TO BMJ OPEN

10th JUNE 2023

[1] Department of Experimental Psychology, University of Oxford, Oxford, UK.

[2] Oxford Health NHS Foundation Trust, Oxford, UK.

[3] Health Economics Research Centre, Nuffield Department of Population Health, Oxford, UK.

[4] The McPin Foundation, London, UK.

[5] Oxford Primary Care Clinical Trials Unit, Nuffield Department of Primary care Health Sciences, University of Oxford, Oxford, UK.

* Address for correspondence: Professor Daniel Freeman, Oxford Cognitive Approaches to Psychosis, Department of Experimental Psychology, University of Oxford, Radcliffe Observatory Quarter, Oxford, UK, OX2 6GG. Email: daniel.freeman@psy.ox.ac.uk

ABSTRACT

Introduction: The confidence of young people diagnosed with psychosis is often low. Positive self-beliefs may be few and negative self-beliefs many. A sense of defeat and failure is common. Young people often withdraw from many aspects of everyday life. Psychological wellbeing is lowered. Psychological techniques can improve self-confidence, but a shortage of therapists means that very few patients ever receive such help. Virtual reality (VR) offers a potential route out of this impasse. By including a virtual coach, treatment can be automated. As such, delivery of effective therapy is no longer reliant on the availability of therapists. With young people with lived experience, we have developed a staff-assisted automated VR therapy to improve positive self-beliefs (Phoenix). The treatment is based on established cognitive behavioural therapy (CBT) and positive psychology techniques. A case series indicates that this approach may lead to large improvements in positive self-beliefs and psychological wellbeing. We now aim to conduct the first randomised controlled evaluation of Phoenix VR.

Methods and analysis: 80 patients with psychosis, aged between 16 and 30 years old and with low levels of positive self-beliefs, will be recruited from National Health Service (NHS) secondary care services. They will be randomised (1:1) to the Phoenix VR self-confidence therapy added to treatment as usual or treatment as usual. Assessments will be conducted at 0, 6 (post-treatment), and 12 weeks by a researcher blind to allocation. The primary outcome is positive self-beliefs at 6 weeks rated with the Oxford Positive Self Scale (OxPos). The secondary outcomes are psychiatric symptoms, activity levels, and quality of life. All main analyses will be intention-to-treat.

Ethics and dissemination: The trial has received ethical approval from the NHS Health Research Authority (22/LO/0273). A key output will be a high-quality VR treatment for patients to improve self-confidence and psychological wellbeing.

Registration: ISRCTN10250113

Strengths and limitation of this study (up to 5 bullet points)

- This is a randomised controlled trial that will provide an estimate of the treatment effect of the addition of the Phoenix VR self-confidence therapy to standard care.
- The primary outcome is a new self-report measure of positive self-beliefs that is strongly associated with psychological well-being.
- The secondary outcomes cover a wide range of psychiatric, functioning, and quality of life domains.
- The trial will not be able to determine the components of the intervention that lead to any clinical benefits.
- The trial is only powered to detect at least moderate effect size improvements in positive self-beliefs.

For peer review only

For peer review only

INTRODUCTION

Patients diagnosed with schizophrenia (and related non-affective psychosis) have often experienced difficult lives (e.g. Varese et al, 2012); may be coping with distressing psychotic experiences, low mood, and anxiety (e.g. Buckley et al, 2009); and can suffer stigma both from themselves and others (e.g. Dickerson et al, 2002). Patients typically view themselves negatively, believe that they are inferior to others, and can be overly self-critical (e.g. Collett et al, 2019). Positive self-views that could counteract such beliefs are often not formed, consolidated, or accessible (e.g. Fowler et al, 2006). The negative self-views, and limited levels of positive self-views, can exacerbate negative affect and psychotic experiences (e.g. Smith et al, 2005). An end result is that often individuals withdraw from many aspects of their lives (e.g. Hooley, 2010). They may become inactive and isolated (e.g. Strassnig et al, 2021), with further negative consequences for mental and physical health. Overall, levels of psychological well-being are often very low in patients with psychosis (e.g. Freeman et al, 2019). We set out to develop a potentially scalable intervention that can build up positive self-beliefs and hence psychological wellbeing. Our aim is to arrest this downward spiral as soon as possible by focussing on patients at the early stages of psychosis.

Psychological techniques delivered in face-to-face therapy, and drawing on CBT and positive psychology (Fennell, 1998; Seligman et al, 2005; Freeman & Freeman, 2012), can improve self-confidence. In a pilot randomised controlled trial of six sessions of face-to-face therapy with patients with current persecutory delusions in the context of a diagnosis of psychosis, we showed that it is possible to achieve large improvements in positive beliefs about the self, social comparison, and psychological well-being (Freeman et al, 2014). However, very few patients receive such psychological therapy. There is a shortage of psychological therapists for patients with schizophrenia. Even fewer therapists have the time to take patients into real-world situations where the most important work is conducted: building up self-confidence through direct experience. Our potential solution is to use immersive technology. Virtual reality (VR) therapy involves wearing a headset and interacting with computer-generated simulations in carefully selected scenarios. The significant advantages of VR are that: it allows users repeatedly to experience therapeutically beneficial simulations; learning has been demonstrated to transfer from the virtual environment to the real world; the therapy can be made compelling and entertaining; and treatment can be automated within VR, removing reliance on the availability of therapists.

In a user-centred design process with young people with lived experience of psychosis we designed a VR therapy to improve positive self-beliefs (Phoenix VR) (Rosebrock et al, in prep). The treatment built upon our face-to-face therapy. The VR therapy had high usability ratings. The treatment is programmed to run on a stand-alone VR consumer headset that can be left with patients. A staff member then has up to six sessions with the patient to introduce the VR therapy and help the person apply the learning to the real

For peer review only

world. Patients can have these sessions at home or in the clinic. An initial proof of concept case series test of Phoenix VR therapy was conducted (Freeman et al, in prep). Twelve young patients diagnosed with psychosis received Phoenix. There were large improvements in positive beliefs about the self and psychological wellbeing after the intervention. However this evaluation did not include a control group. As such, improvements could have been a consequence of natural recovery. A randomised controlled evaluation of Phoenix VR self-confidence therapy is now required.

Aims and hypotheses

Our primary research question is: Does Phoenix VR self-confidence therapy added to treatment as usual, compared to treatment as usual alone, lead to a post-treatment improvement in levels of positive self-beliefs for patients at the early stages of psychosis attending NHS mental health services?

Our primary hypothesis is that:

1. Compared to treatment as usual, Phoenix VR self-confidence therapy added to treatment as usual will improve levels of positive self-beliefs (end of treatment).

Our secondary hypotheses are:

1. Compared to treatment as usual, Phoenix VR self-confidence therapy added to treatment as usual will improve social comparison and psychological well-being (end of treatment).

2. Compared to treatment as usual, Phoenix VR self-confidence therapy added to treatment as usual will reduce depression, negative self-beliefs, hopelessness, anhedonia, anxiety, and paranoia and increase perceptions of recovery, meaningful activity, and quality of life (end of treatment).

3. Treatment effects will be maintained at follow-up (three months).

We will also collect data on satisfaction, side effects, and health economic variables.

For peer review only

METHODS AND ANALYSIS

Trial design and flow chart

The design is a parallel group, superiority, single-blind, randomised controlled trial to test the effects of Phoenix VR therapy when added to usual care. The trial will be run from a single centre, with up to four NHS mental health trusts participating (Oxford Health NHS Foundation Trust; Berkshire Healthcare NHS Foundation Trust; Northamptonshire Healthcare NHS Foundation Trust; Central and North West London NHS Foundation Trust (Milton Keynes)). Treatment as usual will be measured but remain unchanged in both groups. Assessments will be conducted at 0, 6, and 12 weeks. Assessments will be conducted in person or online by research assistants blind to group allocation. A summary of the trial design can be seen in Figure 1. The trial is prospectively registered with the ISRCTN registry: ISRCTN10250113. There is a Data Monitoring and Ethics Committee (DMEC).

Insert Figure 1 about here

Randomisation, blinding, and code-breaking

Participants will be randomised after completing the baseline assessment and allocated to one of the trial arms using a 1:1 allocation ratio. Randomisation will be carried out by a validated online system provided by Sealed Envelope (www.sealedenvelope.com) and using a permuted blocks algorithm, with randomly varying block size.

The research assessors will be blinded to group allocation, but the patients and staff member present will not (they cannot be blinded to whether psychological intervention is delivered or received). If an allocation is revealed between assessment sessions, this will be logged by the trial coordinator and re-blinding will occur using another assessor.

For peer review only

Participants

Participants will be NHS patients diagnosed with non-affective psychosis, with low positive self-beliefs, and aged 16-30 years old. Participants will be recruited principally by seeking referrals to the trial from the relevant clinical teams in the participating mental health trusts (early intervention services, adult community mental health teams, and inpatient units). Patients interested in taking part will then be approached by the research team with the approval of the clinical team, given information about the trial, and screening conducted. All suitable patients will be given at least 24 hours to consider taking part in the trial. Our Lived Experience Advisory Panel (LEAP) has emphasised the importance of patients being able to self-initiate referral. This minimises the chance that particular patients miss out on trials because they are overlooked by clinical teams or their clinician was absent from a referral meeting. However, in all instances we will seek to confirm that a member of the clinical team gives approval for a patient to enter the trial and to complete the necessary screening of eligibility and risk status. Written informed consent will be obtained from all participants.

The inclusion criteria are: Participant is willing and able to give informed consent for participation in the trial; aged between 16 years and 30 years old; attending NHS mental health services for treatment of psychosis; primary clinical diagnosis of non-affective psychosis (schizophrenia, schizoaffective disorder, delusional disorder, or psychosis NOS); low levels of positive self-beliefs indicated by a score of 50 or lower on the Oxford Positive Self Scale (Freeman et al, 2023); stable medication for at least one month and no planned significant changes at the outset of participation.

The exclusion criteria are: a primary diagnosis of another mental health condition (e.g. substance use disorder) that would be the first clinical priority to treat; current engagement in any other intensive individual psychological therapy; in forensic settings or Psychiatric Intensive Care Unit (PICU); command of spoken English inadequate for engaging in the VR therapy; photosensitive epilepsy or significant visual, auditory, or balance impairment that would make use of VR inappropriate; significant learning difficulties that would prevent the completion of assessments. A participant may also be excluded if there is another factor (for example, current active suicidal plans) which, in the judgement of the investigator, would preclude the participant from providing informed consent or from safely engaging with the trial procedures. Reason for exclusions will be recorded.

For peer review only

Assessments

The primary outcome is the total score on the Oxford Positive Self Scale (Freeman et al, 2023). This is a 24-item self-report scale assessing four types of positive self-belief: mastery, strength, enjoyment, and character. It was developed with a representative sample of 2,500 members of the UK population, with further validation in another three thousand individuals. It has a high correlation with psychological wellbeing (r=0.79). The psychometrics of the scale are excellent.

Secondary outcomes are assessments of social comparison, specifically the Social Comparison Scale (Allan & Gilbert, 1995), and psychological wellbeing, using the Warwick-Edinburgh Wellbeing Scale (Tennant et al, 2007). Negative and positive self-beliefs will also be assessed with the Brief Core Schema Scale – Negative and Positive Self subscales (Fowler et al, 2006) and the Everyday Confidence Scale (Waite et al, in prep). Depression will be assessed with the Patient Health Questionnaire -9 (Kroenke et al, 2009), hopelessness with the Beck Hopelessness Scale (Beck & Steer, 1988), anhedonia with the Temporal Experience of Pleasure Scale – anticipatory pleasure (Gard et al, 2006), anxiety with the GAD-7 (Spitzer et al, 2006), and paranoia with the Revised Green et al Paranoid Thoughts Scale (Freeman et al, 2019). We will also assess perceptions of recovery with the Questionnaire of Process of Recovery (QPR) (Law et al, 2014), meaningful activity with a time budget (Jolley et al, 2006), and quality of life with the EQ-5D-5L (Herdman et al, 2011) and the ReQol (Keetharuth et al, 2018). Service use data will be collected with the Modified Client Service Receipt Inventory (Beecham & Knapp, 1992). For patients in the Phoenix VR therapy arm, the staff member will collect end-of-treatment assessments of satisfaction, using the Modified Client Satisfaction Questionnaire (Attkisson & Zwick, 1982; Attkisson & Greenfield, 1999), and side effects, via the Oxford – VR Side Effects Scale (Freeman et al, 2022). A summary of the measures is provided in Table 1.

Insert Table 1 about here

The VR psychological treatment

The treatment being tested is Phoenix VR self-confidence therapy, which is a virtual reality application recommended for adults (16+) attending psychosis services who have low levels of positive self-beliefs.

For peer review only

This software is intended to increase positive self-beliefs. The treatment was designed by our team at the University of Oxford, with young people with lived experience taking part in the design process. The treatment was programmed by the University of Oxford. Phoenix is a UKCA marked, Class I medical device (standalone software as a medical device). The Phoenix software application is composed of a set of virtual environments, including different scenes created using 3D models, ambient audio, and 3D virtual characters, with animations and speech. The environments are driven by source code which handles the logic of the program, the behaviour of the computer characters, as well as the user interaction and data storage. The software is built using Unity (Unity Technologies©). Unity acts as a render engine, displaying the virtual environments to the user through the headset. The application runs through the Unity software application on a Meta Quest 2 VR Headset.

The primary treatment goal is to help young people who have been given a diagnosis of schizophrenia (or related condition) to increase their psychological wellbeing by building up positive self-beliefs. Patients can keep the headset for six weeks in order to use Phoenix on their own. The treatment is supported by a staff member over six sessions. In the first meeting the staff member introduces Phoenix and helps the person try it out. The staff member also helps the person set real-world goals and between-session tasks to improve confidence, and reviews progress. VR does not need to be used in the subsequent in-person meetings since the patient is able to use it at home but most sessions to date have included approximately twenty minutes within VR. Phoenix is used to spark positive self-beliefs that are then consolidated via real-world activities.

The six key mechanisms of action within Phoenix are to: build vivid memories of successful and rewarding interactions; help identify strengths; learn to tolerate fearful emotions and still succeed; learn to appraise situations more positively and with more self-kindness; develop the ability to savour positive experiences and connect with other people; and increase engagement in meaningful activities. Phoenix has three main therapeutic areas within VR for the user: creating a sense of achievement and mastery to develop the belief ‘I can make a difference’; succeeding in challenging situations to develop the belief ‘I can do this’; and engaging in pleasurable activities to develop the belief ‘I can enjoy things’. The achievement and mastery scenario is set in a community garden, with ten tasks relating to the care of plants, animals, and a farmhouse. The challenging situation is a TV studio, with the user having to speak to camera in front of an audience. There are ten levels of difficulty. The pleasurable experiences are set near a lake by a forest and tasks include relaxation exercises and games. A virtual coach (called Farah) guides the participant in the best way to think, feel, and respond. Farah first meets users in her glass-fronted office that overlooks mountains, and provides instructions throughout the programme. Users choose which VR scenarios they wish to complete, and can repeat activities as often as they would like. Pictures of the Phoenix scenarios are provided in Figure 2.

For peer review only

Insert Figure 2 about here

Control condition

Participants who are allocated to the control arm will continue to receive their usual care. No additional interventions will be offered by the research team. Treatment as usual for the participants within this trial will typically consist of prescription of psychiatric medications and meetings with a mental health practitioner. Treatment as usual will vary across individuals. We will collect detailed data on treatment as usual.

Adverse events

A trial standard operational procedure has been written for adverse events. We will record the occurrence of any serious adverse events (SAE) reported to us and also check each patient's medical notes at the end of their participation in the trial. An adverse event is defined by the ISO14155:2011 guidelines for medical device trials as serious if it: a) results in death or, b) is a life-threatening illness or injury or, c) requires hospitalisation or prolongation of existing hospitalisation or, d) results in persistent or significant disability or incapacity or, e) medical or surgical intervention is required to prevent any of the above, f) leads to foetal distress, foetal death or consists of a congenital anomaly or birth defect or, g) is otherwise considered medically significant by the investigator.

Life threatening in the definition of a serious adverse event (SAE) refers to an event in which the subject was at risk of death at the time of the event; it does not refer to an event that hypothetically might have caused death if it were more severe. A planned hospitalization for a pre-existing condition, without a serious deterioration in health, is not considered to be a serious adverse event.

The sorts of serious adverse events that can typically happen to this participant group include: deaths, suicide attempts, serious violent incidents, and admissions to hospital. SAEs are recorded using a Serious

Adverse Event Report Form. The relationship between the investigational medical device or other research procedure and the occurrence of each serious adverse event will be assessed and categorised. The investigators will use clinical judgement to determine the relationship. Alternative causes, such as natural history of the participant's underlying condition, concomitant therapy, other risk factors etc. will be considered. The study team will make an initial assessment of whether the SAE is potentially related to the device or trial procedures and report to the regulatory authorities within the appropriate timescales. The decision about relatedness will be reviewed by the DMEC chair in the first instance.

We will also record adverse events that are not serious. This would include any adverse device effects from the VR treatment, including those resulting from insufficient or inadequate instructions for use, deployment, installation, or operation, or any malfunction of the software. It also includes any event resulting from user error or intentional misuse.

For peer review only

Analysis

A full statistical analysis plan will be approved before any analysis. We will report data in line with the CONSORT 2010 Statement (Schulz et al, 2010) showing attrition rates and loss to follow-up. The primary analyses will be carried out using the intention-to-treat principle. That is, after randomisation, participants will be analysed according to their allocated intervention arm irrespective of what intervention they actually receive, and with data available from all participants included in the analysis including those who do not complete therapy. The outcome analyses will be conducted by statisticians in the University of Oxford Primary Care Clinical Trials Unit.

Baseline variables will be presented by randomised group using frequencies (with percentages) for binary and categorical variables and means (and standard deviations) or medians (with lower and upper quartiles) for continuous variables, along with minimum and maximum values and counts of missing values. There will be no tests of statistical significance nor confidence intervals for differences between groups on any baseline variables. There will be no planned interim analysis for efficacy or futility on the primary outcome.

We will test the primary hypothesis for between-group difference in the primary outcome using a linear mixed effects model which models the response at 6 weeks and 12 weeks, with baseline outcome measure and treatment assignment as fixed effects, with a patient specific random intercept. An interaction between time and randomised group will be fitted as a fixed effect to allow estimation of treatment effect at all time points. The linear mixed effects model will account for missing data assuming data are missing-at-random (MAR). Standard residual diagnostics will be assessed for the appropriateness of the model.

P<0.05 will be used as the level of statistical significance. Similar mixed effect models will be used to analyse secondary outcomes. Treatment differences estimated from linear mixed effects models will additionally be reported as standardised mean differences (mean group difference divided by whole group SD at baseline).

The sample size of 80 patients takes into consideration a maximum attrition rate of 10%, and provides 80% power to detect a moderate difference (d=0.67) in positive self-beliefs, from randomisation to 6 weeks at a 5% level of significance (2-sided).

For peer review only

Ethics and dissemination

The trial has received Health Research Authority (HRA) approval (IRAS 312539). The trial received ethical approval from the NHS London - Harrow Research Ethics Committee (22/LO/0273). The results of the trial will be published in a peer-reviewed journal and made open access. De-identified participant data will be available in anonymised form on reasonable request, subject to review and contract with the University of Oxford, following the publication of results.

Patient and Public Involvement

PPI has been supported by the McPin Foundation, a charity that aims to put the lived experience perspective at the heart of mental health research. A Patient Advisory Group (PAG), comprising young people who have been diagnosed with psychosis, has helped design the VR therapy, try prototypes, and contributed to the development of the primary outcome measure.

Competing interests

DF and JF are founders of Oxford VR, a University of Oxford spin-out company. Oxford VR has not been involved in the Phoenix project.

Authors' contributions

DF is the chief investigator, conceived the project, secured the funding, had overall responsibility for the treatment design and the trial design, and drafted the trial protocol. LRo, JF, FW, AR, ALM, RW, MB, and LRi contributed to the treatment design. AR, ALM, RW, MB, and LRi conducted the programming. LRo is co-ordinating the trial. TK is responsible for lived experience involvement. L-MY is responsible for the statistical analysis. JL will be responsible for health economic data. All authors commented on the trial protocol.

Funding

International Foundation (https://www.if-internationalfoundation.org/en/home-page) award to DF. The project is also supported by the NIHR Oxford Health Biomedical Research Centre. DF is an NIHR Senior Investigator and is supported by the NIHR Oxford Health Biomedical Research Centre. FW is supported by a Wellcome Trust Doctoral Fellowship. The views expressed are those of the authors and not necessarily those of the NHS, the NIHR or the Department of Health.

For peer review only

REFERENCES

Allan, S., Gilbert, P., (1995). Social comparison scale: psychometric properties and relationship to psychopathology. Personality and Individual Differences, 19, 293-299.

Attkisson, C.C. & Greenfield, T.K. (1999). The UCSF Client Satisfaction Scales: I. The Client Satisfaction Questionnaire-8. Pp 1333-1346. In The use of psychological testing for treatment, planning and outcome assessments (Ed. M.E. Maruish). Second edition. Mahwah, NJ: Lawrence Erlbaum Associates.

Attkisson, C.C. & Zwick, R. (1982). The client satisfaction questionnaire. Evaluation and Program Planning, 5, 233–237.

Beck, A. T., & Steer, R. A. (1988). Manual for the Beck Hopelessness Scale. San Antonio, TX: Psychological Corp.

Beecham, J. & Knapp, M. (1992). Costing psychiatric interventions. In Measuring Mental Health Needs (Eds. G Thornicroft, CR Brewin, JK Wing). pp 163-184. London: Gaskell.

Buckley, P. F., Miller, B. J., Lehrer, D. S., & Castle, D. J. (2009). Psychiatric comorbidities and schizophrenia. Schizophrenia bulletin, 35(2), 383-402.

Collett, N., Pugh, K., Waite, F., & Freeman, D. (2016). Negative cognitions about the self in patients with persecutory delusions: An empirical study of self-compassion, self-stigma, schematic beliefs, self-esteem, fear of madness, and suicidal ideation. Psychiatry research, 239, 79-84.

Dickerson, F. B., Sommerville, J., Origoni, A. E., Ringel, N. B., & Parente, F. (2002). Experiences of stigma among outpatients with schizophrenia. Schizophrenia bulletin, 28(1), 143-155.

Fennell, M. J. (1998). Cognitive therapy in the treatment of low self-esteem. Advances in Psychiatric Treatment, 4(5), 296-304.

Fowler, D., Freeman, D., Smith, B., Kuipers, E., Bebbington, P., Bashforth, H. et al. (2006). The Brief Core Schema Scales (BCSS): psychometric properties and associations with paranoia and grandiosity in non-clinical and psychosis samples. Psychological Medicine, 36, 749-759.

Freeman, D., Freeman, J., Ahmed, M., Haynes, P., Beckwith, H., Rovira, A., Miguel, A.L., Ward, R., Bousfield, M., Riffiod, L., Kabir, T., Waite, F., & Rosebrock, L. (in prep). Automated VR therapy for improving positive self-beliefs and psychological wellbeing in young patients with psychosis: a proof of concept evaluation of Phoenix VR self-confidence therapy.

Freeman, D., Loe, B.S., Kingdon, D., Startup, H., Molodynski, A., Rosebrock, L., Brown, P., Sheaves, B., Waite, F., & Bird, J.C. (2019). The revised Green et al., Paranoid Thoughts Scale (R-GPTS): psychometric properties, severity ranges, and clinical cut-offs. Psychological Medicine, 51, 244-253.

Freeman, D., Pugh, K., Dunn, G., Evans, N., Sheaves, B., Waite, F., Černis, E., Lister, R., & Fowler, D. (2014). An early Phase II randomised controlled trial testing the effect on persecutory delusions of using CBT to reduce negative cognitions about the self: the potential benefits of enhancing self confidence. Schizophrenia research, 160, 186-192.

For peer review only

Freeman, D., Rosebrock, L., Loe, B.S., Saidel, S., Freeman, J., & Waite, F. (2023). The Oxford Positive Self Scale: psychometric development of an assessment of cognitions associated with psychological well-being. Psychological Medicine, pp.1-9.

Freeman, D., Rosebrock, L., Waite, F., Loe, B.S., Kabir, T., Petit, A., Dudley, R., Chapman, K., Morrison, A., O'Regan, E., Aynsworth, C., Jones, J., Murphy, E., Powling, R., Peel, H., Walker, H., Bryne, R., Freeman, J., Rovira, A., Galal, U., Yu, L-M., Clark, D.M., & Lambe. S. (2022). Virtual reality (VR) therapy for patients with psychosis: satisfaction and side effects. Psychological Medicine. DOI: https://doi.org/10.1017/S0033291722001167

Freeman, D., Taylor, K. M., Molodynski, A., & Waite, F. (2019). Treatable clinical intervention targets for patients with schizophrenia. Schizophrenia Research, 211, 44-50.

Gard, D. E., Gard, M. G., Kring, A. M., & John, O. P. (2006). Anticipatory and consummatory components of the experience of pleasure: A scale development study. Journal of Research in Personality, 40(6), 1086-1102

Herdman, M., Gudex, C., Lloyd, A., Janssen, M., Kind, P., Parkin, D., Bonsel, G., & Badia, X. (2011). Development and preliminary testing of the new five-level version of EQ-5D (EQ-5D-5L). Quality of Life Research, 20, 1727-1736.

Hooley, J. M. (2010). Social factors in schizophrenia. Current directions in psychological science, 19(4), 238-242.

Jolley, S., Garety, P. A., Ellett, L., Kuipers, E., Freeman, D., Bebbington, P. E., Fowler, D. G., & Dunn, G. (2006). A validation of a new measure of activity in psychosis. Schizophrenia Research, 85(1–3), 288–295.

Keetharuth, A., Brazier, J., Connell, J., Bjorner, J., Carlton, J., Taylor Buck, E., Ricketts, T., McKendrick, K., Browne, J., Croudace, T., & Barkham, M. (2018). Recovering Quality of Life (ReQoL): a new generic self-reported outcome measure for use with people experiencing mental health difficulties. British Journal of Psychiatry, 212, 42-49.

Kroenke, K., Spitzer, R., & Williams, J. (2001). The PHQ-9. Journal of General Internal Medicine, 16, 606-613.

Law, H., Neil, S., Dunn, G., & Morrison, A. (2014). Psychometric properties of the Questionnaire about the Process of Recovery (QPR). Schizophrenia Research, 156, 184-189.

Schulz, K. F., Altman, D. G., & Moher, D. (2010). CONSORT 2010 Statement: updated guidelines for reporting parallel group randomised trials. BMC Medicine, 8(1), 18. ttps://doi.org/10.1186/1741-7015-8-18

Seligman, M. E. P., Steen, T. A., Park, N., & Peterson, C. (2005). Positive psychology progress: Empirical validation of interventions. American Psychologist, 60, 410–421.

Smith, B., Fowler, D.G., Freeman, D., Bebbington, P., Bashforth, H., Garety, P., Dunn, G., & Kuipers, E. (2006). Emotion and psychosis: links between depression, self-esteem, negative schematic beliefs and delusions and hallucinations. Schizophrenia research, 86, 181-188.

For peer review only

Spitzer, R. L., Kroenke, K., Williams, J. B. W., & Löwe, B. (2006). A brief measure for assessing generalized anxiety disorder: The GAD-7. Archives of Internal Medicine, 166, 1092-1097.

Strassnig, M. T., Harvey, P. D., Miller, M. L., Depp, C. A., & Granholm, E. (2021). Real world sedentary behavior and activity levels in patients with schizophrenia and controls: An ecological momentary assessment study. Mental health and physical activity, 20, 100364.

Tennant, R., Hiller, L., Fishwick, R., Platt, S., Joseph, S., Weich, S. et al. (2007). The Warwick-Edinburgh Mental Well-being Scale (WEMWBS): development and UK validation. Health and Quality of Life Outcomes, 5, 63-80.

Varese, F., Smeets, F., Drukker, M., Lieverse, R., Lataster, T., Viechtbauer, W., Read, J., Van Os, J. & Bentall, R.P. (2012). Childhood adversities increase the risk of psychosis: a meta-analysis of patient-control, prospective-and cross-sectional cohort studies. Schizophrenia bulletin, 38, 661-671.

For peer review only

Figure 1. Trial flow diagram

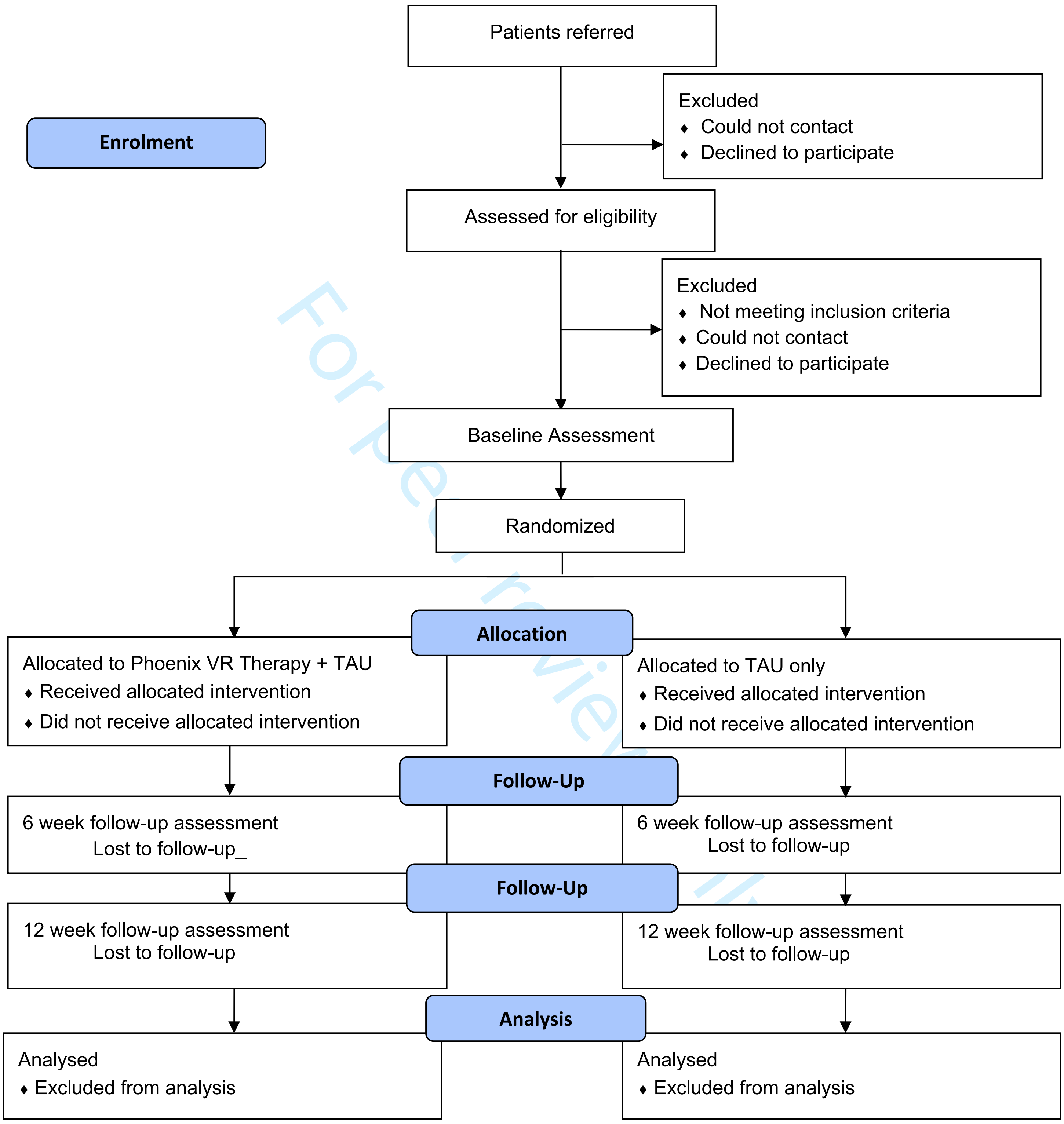

Figure 2. Pictures of Phoenix VR self-confidence therapy

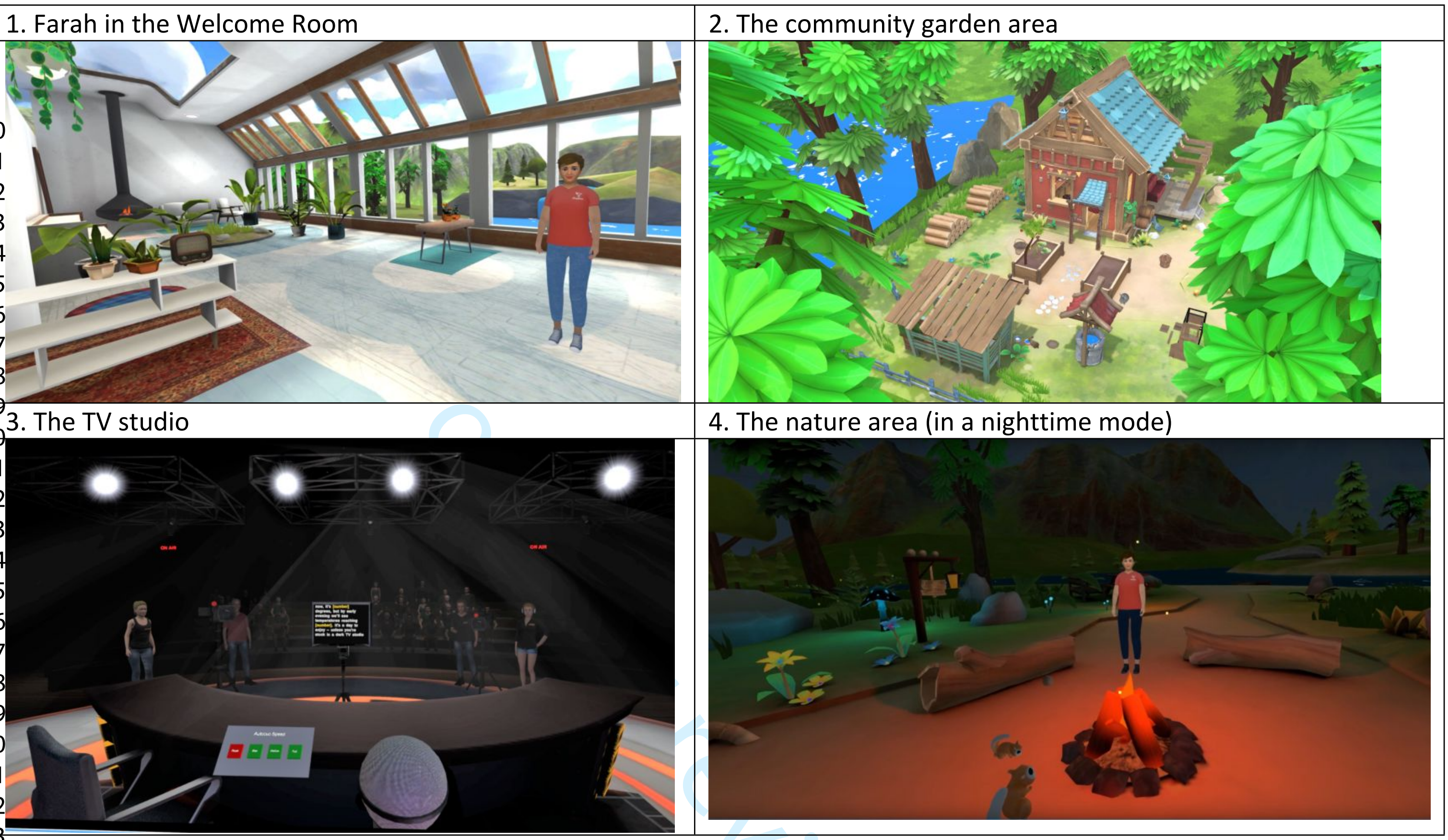

Table 1. Summary of objectives and assessment measures

	Objectives	Outcome Measures
Primary	Test whether the virtual reality treatment leads to improvement in positive self-beliefs.	Oxford Positive Self Scale (OxPos)
Secondary	Test whether the VR therapy improves social comparison and psychological well-being.	Social Comparison Scale, Warwick-Edinburgh Wellbeing Scale
	Test whether the VR therapy leads to reductions in depression, negative self-beliefs, everyday self-confidence, hopelessness, anhedonia, anxiety, and paranoia and increases in perceptions of recovery, meaningful activity, and quality of life.	Patient Health Questionnaire -9, Brief Core Schema Scale, Everyday Confidence Scale, Beck Hopelessness Scale, Temporal Experience of Pleasure Scale, GAD-7, Revised Green et al Paranoid Thoughts Scale, Questionnaire of Process of Recovery, time budget, EQ-5D-5L, ReQol.
	Assess patient satisfaction with the VR therapy.	Modified version of the Client Satisfaction Questionnaire.
	Assess side effects of the VR therapy	Oxford – VR Side Effects Scale

For peer review only

BMJ Open

A Randomised Controlled Trial of Automated VR Therapy to Improve Positive Self-Beliefs and Psychological Wellbeing in Young People Diagnosed with Psychosis: Study Protocol for the Phoenix VR Self-Confidence Therapy Trial

Journal:	*BMJ Open*
Manuscript ID	bmjopen-2023-076559.R1
Article Type:	Protocol
Date Submitted by the Author:	17-Oct-2023
Complete List of Authors:	Freeman, Daniel; University of Oxford, Department of Psychiatry<br>Freeman, Jason; University of Oxford, Experimental Psychology<br>Rovira, Aitor; University of Oxford, Department of Experimental Psychology<br>Miguel, Andre Lages; University of Oxford, Department of Experimental Psychology<br>Ward, Rupert; University of Oxford, Department of Experimental Psychology<br>Bousfield, Matthew; University of Oxford, Department of Experimental Psychology<br>Riffiod, Ludovic; University of Oxford, Department of Experimental Psychology<br>Leal, Jose; University of Oxford, Nuffield Department of Population Health<br>Kabir, Thomas; The McPin Foundation<br>Yu, Ly-Mee; University of Oxford, Department of Primary Care Health Sciences<br>Beckwith, Helen; University of Oxford, Department of Experimental Psychology<br>Waite, Felicity; University of Oxford Department of Psychiatry,<br>Rosebrock, Laina; University of Oxford, Department of Experimental Psychology
**Primary Subject Heading**:	Mental health
Secondary Subject Heading:	Evidence based practice
Keywords:	Schizophrenia & psychotic disorders < PSYCHIATRY, Virtual Reality, Clinical Trial

SCHOLARONE™
Manuscripts

A Randomised Controlled Trial of Automated VR Therapy to Improve Positive Self-Beliefs and Psychological Wellbeing in Young People Diagnosed with Psychosis: Study Protocol for the Phoenix VR Self-Confidence Therapy Trial

Daniel Freeman[1,2]*, Jason Freeman[1], Aitor Rovira[1,2], Andre Lages Miguel[1], Rupert Ward[1,2], Matthew Bousfield[1], Ludovic Riffiod[1], José Leal[3], Thomas Kabir[1,4], Ly-Mee Yu[5], Helen Beckwith[1,2], Felicity Waite[1,2], Laina Rosebrock[1,2]

For peer review only

REVISION TO BMJ OPEN

14th OCTOBER 2023

[1] Department of Experimental Psychology, University of Oxford, Oxford, UK.

[2] Oxford Health NHS Foundation Trust, Oxford, UK.

[3] Health Economics Research Centre, Nuffield Department of Population Health, Oxford, UK.

[4] Department of Psychiatry, University of Oxford, Oxford, UK.

[5] Oxford Primary Care Clinical Trials Unit, Nuffield Department of Primary care Health Sciences, University of Oxford, Oxford, UK.

* Address for correspondence: Professor Daniel Freeman, Oxford Cognitive Approaches to Psychosis, Department of Experimental Psychology, University of Oxford, Radcliffe Observatory Quarter, Oxford, UK, OX2 6GG. Email: daniel.freeman@psy.ox.ac.uk

ABSTRACT

Introduction: The confidence of young people diagnosed with psychosis is often low. Positive self-beliefs may be few and negative self-beliefs many. A sense of defeat and failure is common. Young people often withdraw from many aspects of everyday life. Psychological wellbeing is lowered. Psychological techniques can improve self-confidence, but a shortage of therapists means that very few patients ever receive such help. Virtual reality (VR) offers a potential route out of this impasse. By including a virtual coach, treatment can be automated. As such, delivery of effective therapy is no longer reliant on the availability of therapists. With young people with lived experience, we have developed a staff-assisted automated VR therapy to improve positive self-beliefs (Phoenix). The treatment is based on established cognitive behavioural therapy (CBT) and positive psychology techniques. A case series indicates that this approach may lead to large improvements in positive self-beliefs and psychological wellbeing. We now aim to conduct the first randomised controlled evaluation of Phoenix VR.

Methods and analysis: 80 patients with psychosis, aged between 16 and 30 years old and with low levels of positive self-beliefs, will be recruited from National Health Service (NHS) secondary care services. They will be randomised (1:1) to the Phoenix VR self-confidence therapy added to treatment as usual or treatment as usual. Assessments will be conducted at 0, 6 (post-treatment), and 12 weeks by a researcher blind to allocation. The primary outcome is positive self-beliefs at 6 weeks rated with the Oxford Positive Self Scale (OxPos). The secondary outcomes are psychiatric symptoms, activity levels, and quality of life. All main analyses will be intention-to-treat.

Ethics and dissemination: The trial has received ethical approval from the NHS Health Research Authority (22/LO/0273). A key output will be a high-quality VR treatment for patients to improve self-confidence and psychological wellbeing.

Registration: ISRCTN10250113

For peer review only

Strengths and limitation of this study (up to 5 bullet points)

- This is a randomised controlled trial that will provide an estimate of the treatment effect of the addition of the Phoenix VR self-confidence therapy to standard care.
- The primary outcome is a new self-report measure of positive self-beliefs that is strongly associated with psychological well-being.
- The secondary outcomes cover a wide range of psychiatric, functioning, and quality of life domains.
- The trial will not be able to determine the components of the intervention that lead to any clinical benefits.
- The trial is only powered to detect at least moderate effect size improvements in positive self-beliefs.

For peer review only

INTRODUCTION

Patients diagnosed with schizophrenia (and related non-affective psychosis) have often experienced difficult lives [1]; may be coping with distressing psychotic experiences, low mood, and anxiety [2]; and can suffer stigma both from themselves and others [3]. Patients typically view themselves negatively, believe that they are inferior to others, and can be overly self-critical [4]. Positive self-views that could counteract such beliefs are often not formed, consolidated, or accessible [5]. The negative self-views, and limited levels of positive self-views, can exacerbate negative affect and psychotic experiences [6]. An end result is that often individuals withdraw from many aspects of their lives [7]. They may become inactive and isolated [8}, with further negative consequences for mental and physical health. Overall, levels of psychological well-being are often very low in patients with psychosis [9]. We set out to develop a potentially scalable intervention that can build up positive self-beliefs and hence psychological wellbeing. Our aim is to arrest this downward spiral as soon as possible by focussing on patients at the early stages of psychosis.

Psychological techniques delivered in face-to-face therapy, and drawing on CBT and positive psychology (10-12], can improve self-confidence. In a pilot randomised controlled trial of six sessions of face-to-face therapy with patients with current persecutory delusions in the context of a diagnosis of psychosis, we showed that it is possible to achieve large improvements in positive beliefs about the self, social comparison, and psychological well-being [13]. However, very few patients receive such psychological therapy. There is a shortage of psychological therapists for patients with schizophrenia. Even fewer therapists have the time to take patients into real-world situations where the most important work is conducted: building up self-confidence through direct experience. Our potential solution is to use immersive technology. Virtual reality (VR) therapy involves wearing a headset and interacting with computer-generated simulations in carefully selected scenarios. The significant advantages of VR are that: it allows users repeatedly to experience therapeutically beneficial simulations; learning has been demonstrated to transfer from the virtual environment to the real world [14-16]; the therapy can be made compelling and entertaining; and treatment can be automated within VR, removing reliance on the availability of therapists [17].

In a user-centred design process with young people with lived experience of psychosis we designed a VR therapy to improve positive self-beliefs (Phoenix VR) (Rosebrock et al, submitted). The treatment built upon our face-to-face therapy. The VR therapy had high usability ratings. The treatment is programmed to run on a stand-alone VR consumer headset that can be left with patients. A staff member then has up to six sessions with the patient to introduce the VR therapy and help the person apply the learning to the real world. Patients can have these sessions at home or in the clinic. An initial proof of concept case series test

For peer review only - http://bmjopen.bmj.com/site/about/guidelines.xhtml

of Phoenix VR therapy was conducted [18]. Twelve young patients diagnosed with psychosis received Phoenix. There were large improvements in positive beliefs about the self (d=3.0) and psychological wellbeing (d=1.5) after the intervention. However this evaluation did not include a control group. As such, improvements could have been a consequence of natural recovery. A randomised controlled evaluation of Phoenix VR self-confidence therapy is now required.

Aims and hypotheses

Our primary research question is: Does Phoenix VR self-confidence therapy added to treatment as usual, compared to treatment as usual alone, lead to a post-treatment improvement in levels of positive self-beliefs for patients at the early stages of psychosis attending NHS mental health services?

Our primary hypothesis is that:

1. Compared to treatment as usual, Phoenix VR self-confidence therapy added to treatment as usual will improve levels of positive self-beliefs (end of treatment).

Our secondary hypotheses are:

1. Compared to treatment as usual, Phoenix VR self-confidence therapy added to treatment as usual will improve social comparison and psychological well-being (end of treatment).

2. Compared to treatment as usual, Phoenix VR self-confidence therapy added to treatment as usual will reduce depression, negative self-beliefs, hopelessness, anhedonia, anxiety, and paranoia and increase perceptions of recovery, meaningful activity, and quality of life (end of treatment).

3. Treatment effects will be maintained at follow-up (three months).

We will also collect data on satisfaction, side effects, and health economic variables.

METHODS AND ANALYSIS

For peer review only

Trial design and flow chart

The design is a parallel group, superiority, single-blind, randomised controlled trial to test the effects of Phoenix VR therapy when added to usual care. The trial will be run from a single centre, with up to four NHS mental health trusts participating (Oxford Health NHS Foundation Trust; Berkshire Healthcare NHS Foundation Trust; Northamptonshire Healthcare NHS Foundation Trust; Central and North West London NHS Foundation Trust (Milton Keynes)). Treatment as usual will be measured but remain unchanged in both groups. Assessments will be conducted at 0, 6, and 12 weeks. Assessments will be conducted in person or online by research assistants blind to group allocation. A summary of the trial design can be seen in Figure 1. The trial is prospectively registered with the ISRCTN registry: ISRCTN10250113. The University of Oxford is the trial sponsor. There is a Data Monitoring and Ethics Committee (DMEC).

Insert Figure 1 about here

Randomisation, blinding, and code-breaking

Participants will be randomised after completing the baseline assessment and allocated to one of the trial arms using a 1:1 allocation ratio. Randomisation will be carried out by a validated online system provided by Sealed Envelope (www.sealedenvelope.com) and using a permuted blocks algorithm, with randomly varying block size.

The research assessors will be blinded to group allocation, but the patients and staff member present will not (they cannot be blinded to whether psychological intervention is delivered or received). If an allocation is revealed between assessment sessions, this will be logged by the trial coordinator and re-blinding will occur using another assessor.

Participants

For peer review only - http://bmjopen.bmj.com/site/about/guidelines.xhtml

For peer review only

Participants will be NHS patients diagnosed with non-affective psychosis, with low positive self-beliefs, and aged 16-30 years old. Participants will be recruited principally by seeking referrals to the trial from the relevant clinical teams in the participating mental health trusts (early intervention services, adult community mental health teams, and inpatient units). Patients interested in taking part will then be approached by the research team with the approval of the clinical team, given information about the trial, and screening conducted. All suitable patients will be given at least 24 hours to consider taking part in the trial. Our Lived Experience Advisory Panel (LEAP) has emphasised the importance of patients being able to self-initiate referral. This minimises the chance that particular patients miss out on trials because they are overlooked by clinical teams or their clinician was absent from a referral meeting. However, in all instances we will seek to confirm that a member of the clinical team gives approval for a patient to enter the trial and to complete the necessary screening of eligibility and risk status. Written informed consent will be obtained from all participants.

The inclusion criteria are: Participant is willing and able to give informed consent for participation in the trial; aged between 16 years and 30 years old; attending NHS mental health services for treatment of psychosis; primary clinical diagnosis of non-affective psychosis (schizophrenia, schizoaffective disorder, delusional disorder, or psychosis NOS); low levels of positive self-beliefs indicated by a score of 50 or lower on the Oxford Positive Self Scale [19]; stable medication for at least one month and no planned significant changes at the outset of participation.

The exclusion criteria are: a primary diagnosis of another mental health condition (e.g. substance use disorder) that would be the first clinical priority to treat; current engagement in any other intensive individual psychological therapy; in forensic settings or Psychiatric Intensive Care Unit (PICU); command of spoken English inadequate for engaging in the VR therapy; photosensitive epilepsy or significant visual, auditory, or balance impairment that would make use of VR inappropriate; significant learning difficulties that would prevent the completion of assessments. A participant may also be excluded if there is another factor (for example, current active suicidal plans) which, in the judgement of the investigator, would preclude the participant from providing informed consent or from safely engaging with the trial procedures. Reason for exclusions will be recorded.

For peer review only

Assessments

The primary outcome is the total score on the Oxford Positive Self Scale [19]. This is a 24-item self-report scale assessing four types of positive self-belief: mastery, strength, enjoyment, and character. It was developed with a representative sample of 2,500 members of the UK population, with further validation in another three thousand individuals. It has a high correlation with psychological wellbeing (r=0.79). The psychometrics of the scale are excellent, including internal consistency (omega coefficient=0.92).

Secondary outcomes are assessments of social comparison, specifically the Social Comparison Scale [20], and psychological wellbeing, using the Warwick-Edinburgh Wellbeing Scale [21]. Negative and positive self-beliefs will also be assessed with the Brief Core Schema Scale – Negative and Positive Self subscales [5] and the Everyday Confidence Scale [22]. Depression will be assessed with the Patient Health Questionnaire -9 [23], hopelessness with the Beck Hopelessness Scale [24], anhedonia with the Temporal Experience of Pleasure Scale – anticipatory pleasure [25], anxiety with the GAD-7 [26], and paranoia with the Revised Green et al Paranoid Thoughts Scale [27]. We will also assess perceptions of recovery with the Questionnaire of Process of Recovery (QPR) [28], meaningful activity with a time budget [29], and quality of life with the EQ-5D-5L [30] and the ReQol [31]. Service use data will be collected with the Modified Client Service Receipt Inventory [32]. For patients in the Phoenix VR therapy arm, the staff member will collect end-of-treatment assessments of satisfaction, using the Modified Client Satisfaction Questionnaire [33. 34], and side effects, via the Oxford – VR Side Effects Scale [35]. A summary of the measures is provided in Table 1.

Insert Table 1 about here

The VR psychological treatment

The treatment being tested is Phoenix VR self-confidence therapy, which is a virtual reality application recommended for adults (16+) attending psychosis services who have low levels of positive self-beliefs. This software is intended to increase positive self-beliefs. The treatment was designed by our team at the University of Oxford, with young people with lived experience taking part in the design process. The treatment was programmed by the University of Oxford. Phoenix is a UKCA marked, Class I medical device

For peer review only

(standalone software as a medical device). The Phoenix software application is composed of a set of virtual environments, including different scenes created using 3D models, ambient audio, and 3D virtual characters, with animations and speech. The environments are driven by source code which handles the logic of the program, the behaviour of the computer characters, as well as the user interaction and data storage. The software is built using Unity (Unity Technologies©). Unity acts as a render engine, displaying the virtual environments to the user through the headset. The application runs through the Unity software application on a Meta Quest 2 VR Headset.

The primary treatment goal is to help young people who have been given a diagnosis of schizophrenia (or related condition) to increase their psychological wellbeing by building up positive self-beliefs. Patients can keep the headset for six weeks in order to use Phoenix on their own. The treatment is supported by a staff member over six sessions. In the first meeting the staff member introduces Phoenix and helps the person try it out. The staff member also helps the person set real-world goals and between-session tasks to improve confidence, and reviews progress. VR does not need to be used in the subsequent in-person meetings since the patient is able to use it at home but most sessions to date have included approximately twenty minutes within VR. Phoenix is used to spark positive self-beliefs that are then consolidated via real-world activities.

The six key mechanisms of action within Phoenix are to: build vivid memories of successful and rewarding interactions; help identify strengths; learn to tolerate fearful emotions and still succeed; learn to appraise situations more positively and with more self-kindness; develop the ability to savour positive experiences and connect with other people; and increase engagement in meaningful activities. Phoenix has three main therapeutic areas within VR for the user: creating a sense of achievement and mastery to develop the belief 'I can make a difference'; succeeding in challenging situations to develop the belief 'I can do this'; and engaging in pleasurable activities to develop the belief 'I can enjoy things'. The achievement and mastery scenario is set in a community garden, with ten tasks relating to the care of plants, animals, and a farmhouse. Each task is broken down into several steps for the participant to complete. For example, caring for plants includes preparing the soil, planting seeds, watering, and picking the crops. The challenging situation is a TV studio, with the user having to speak to camera in front of an audience. Patients read out a weather forecast presented on an autocue. There are ten levels of difficulty, varying in the size of the audience, the difficulty of the speaking task, and the environment itself (e.g. gradual introduction of cameras and spotlights). The pleasurable experiences are set near a lake by a forest and tasks include relaxation exercises (e.g. savouring, progressive muscle relaxation) and games (e.g. a throwing game, playing on a drum set). A virtual coach (called Farah) guides the participant in the best way to think, feel, and respond. Farah provides instruction, encouragement, and positive reinforcement. Farah first meets users in her glass-fronted office that overlooks mountains, and provides instructions

For peer review only

throughout the programme. Users choose which VR scenarios they wish to complete, and can repeat activities as often as they would like.

Control condition

Participants who are allocated to the control arm will continue to receive their usual care. No additional interventions will be offered by the research team. Treatment as usual for the participants within this trial will typically consist of prescription of psychiatric medications and meetings with a mental health practitioner. Treatment as usual will vary across individuals. We will collect detailed data on treatment as usual.

Adverse events

A trial standard operational procedure has been written for adverse events. We will record the occurrence of any serious adverse events (SAE) reported to us and also check each patient's medical notes at the end of their participation in the trial. An adverse event is defined by the ISO14155:2011 guidelines for medical device trials as serious if it: a) results in death or, b) is a life-threatening illness or injury or, c) requires hospitalisation or prolongation of existing hospitalisation or, d) results in persistent or significant disability or incapacity or, e) medical or surgical intervention is required to prevent any of the above, f) leads to foetal distress, foetal death or consists of a congenital anomaly or birth defect or, g) is otherwise considered medically significant by the investigator.

Life threatening in the definition of a serious adverse event (SAE) refers to an event in which the subject was at risk of death at the time of the event; it does not refer to an event that hypothetically might have caused death if it were more severe. A planned hospitalization for a pre-existing condition, without a serious deterioration in health, is not considered to be a serious adverse event.

The sorts of serious adverse events that can typically happen to this participant group include: deaths, suicide attempts, serious violent incidents, and admissions to hospital. SAEs are recorded using a Serious Adverse Event Report Form. The relationship between the investigational medical device or other research procedure and the occurrence of each serious adverse event will be assessed and categorised. The investigators will use clinical judgement to determine the relationship (including whether the patient reports a link to the study). Alternative causes, such as natural history of the participant's underlying

condition, concomitant therapy, other risk factors etc. will be considered. The study team will make an initial assessment of whether the SAE is potentially related to the device or trial procedures and report to the regulatory authorities within the appropriate timescales. The decision about relatedness will be reviewed by the DMEC chair in the first instance.

We will also record adverse events that are not serious. This would include any adverse device effects from the VR treatment, including those resulting from insufficient or inadequate instructions for use, deployment, installation, or operation, or any malfunction of the software. It also includes any event resulting from user error or intentional misuse.

For peer review only

Analysis

A full statistical analysis plan will be approved before any analysis. We will report data in line with the CONSORT 2010 Statement [36] showing attrition rates and loss to follow-up. The primary analyses will be carried out using the intention-to-treat principle. That is, after randomisation, participants will be analysed according to their allocated intervention arm irrespective of what intervention they actually receive, and with data available from all participants included in the analysis including those who do not complete therapy. The outcome analyses will be conducted by statisticians in the University of Oxford Primary Care Clinical Trials Unit.

Baseline variables will be presented by randomised group using frequencies (with percentages) for binary and categorical variables and means (and standard deviations) or medians (with lower and upper quartiles) for continuous variables, along with minimum and maximum values and counts of missing values. There will be no tests of statistical significance nor confidence intervals for differences between groups on any baseline variables. There will be no planned interim analysis for efficacy or futility on the primary outcome.

We will test the primary hypothesis for between-group difference in the primary outcome using a linear mixed effects model which models the response at 6 weeks and 12 weeks, with baseline outcome measure and treatment assignment as fixed effects, with a patient specific random intercept. An interaction between time and randomised group will be fitted as a fixed effect to allow estimation of treatment effect at all time points. The linear mixed effects model will account for missing data assuming data are missing-at-random (MAR). Standard residual diagnostics will be assessed for the appropriateness of the model. $P<0.05$ will be used as the level of statistical significance. Similar mixed effect models will be used to analyse secondary outcomes. Treatment differences estimated from linear mixed effects models will

additionally be reported as standardised mean differences (mean group difference divided by whole group SD at baseline).

The sample size of 80 patients takes into consideration a maximum attrition rate of 10%, and provides 80% power to detect a moderate difference (d=0.67) in positive self-beliefs, from randomisation to 6 weeks at a 5% level of significance using an independent groups t test (2-sided). Further power is likely to be gained by use of mixed-effects models.

Ethics and dissemination

The trial has received Health Research Authority (HRA) approval (IRAS 312539). The trial received ethical approval from the NHS London - Harrow Research Ethics Committee (22/LO/0273). The results of the trial will be published in a peer-reviewed journal and made open access. De-identified participant data will be available in anonymised form on reasonable request, subject to review and contract with the University of Oxford, following the publication of results.

Patient and Public Involvement

PPI has been supported by the McPin Foundation, a charity that aims to put the lived experience perspective at the heart of mental health research. A Patient Advisory Group (PAG), comprising young people who have been diagnosed with psychosis, has helped design the VR therapy, try prototypes, and contributed to the development of the primary outcome measure.

Data sharing

Requests - accompanied by a study summary - for sharing of de-identified data will be considered by the Chief Investigator and team. The intent is to share data for reasonable requests. Data will be made available to external researchers subject to the constraints of the consent under which data were collected, with an appropriate data sharing agreement, and after publication of the main study report.

Competing interests

For peer review only

DF and JF are founders of Oxford VR, a University of Oxford spin-out company. Oxford VR has not been involved in the Phoenix project.

Authors' contributions

DF is the chief investigator, conceived the project, secured the funding, had overall responsibility for the treatment design and the trial design, and drafted the trial protocol. LRo, JF, FW, AR, ALM, RW, MB, and LRi contributed to the treatment design. AR, ALM, RW, MB, and LRi conducted the programming. LRo and HB are co-ordinating the trial. TK is responsible for lived experience involvement. L-MY is responsible for the statistical analysis. JL will be responsible for health economic data. All authors commented on the trial protocol.

Funding

International Foundation (https://www.if-internationalfoundation.org/en/home-page) award to DF. The project is also supported by the NIHR Oxford Health Biomedical Research Centre. DF is an NIHR Senior Investigator and is supported by the NIHR Oxford Health Biomedical Research Centre. FW is supported by a Wellcome Trust Doctoral Fellowship. The views expressed are those of the authors and not necessarily those of the NHS, the NIHR or the Department of Health.

For peer review only

REFERENCES

1. Varese, F., Smeets, F., Drukker, M., Lieverse, R., Lataster, T., Viechtbauer, W., Read, J., Van Os, J. & Bentall, R.P. (2012). Childhood adversities increase the risk of psychosis: a meta-analysis of patient-control, prospective-and cross-sectional cohort studies. Schizophrenia bulletin, 38, 661-671.

2. Buckley, P. F., Miller, B. J., Lehrer, D. S., & Castle, D. J. (2009). Psychiatric comorbidities and schizophrenia. Schizophrenia bulletin, 35(2), 383-402.

3. Dickerson, F. B., Sommerville, J., Origoni, A. E., Ringel, N. B., & Parente, F. (2002). Experiences of stigma among outpatients with schizophrenia. Schizophrenia bulletin, 28(1), 143-155.

4. Collett, N., Pugh, K., Waite, F., & Freeman, D. (2016). Negative cognitions about the self in patients with persecutory delusions: An empirical study of self-compassion, self-stigma, schematic beliefs, self-esteem, fear of madness, and suicidal ideation. Psychiatry research, 239, 79-84.

5. Fowler, D., Freeman, D., Smith, B., Kuipers, E., Bebbington, P., Bashforth, H. et al. (2006). The Brief Core Schema Scales (BCSS): psychometric properties and associations with paranoia and grandiosity in non-clinical and psychosis samples. Psychological Medicine, 36, 749-759.

6. Smith, B., Fowler, D.G., Freeman, D., Bebbington, P., Bashforth, H., Garety, P., Dunn, G., & Kuipers, E. (2006). Emotion and psychosis: links between depression, self-esteem, negative schematic beliefs and delusions and hallucinations. Schizophrenia research, 86, 181-188.

7. Hooley, J. M. (2010). Social factors in schizophrenia. Current directions in psychological science, 19(4), 238-242.

8. Strassnig, M. T., Harvey, P. D., Miller, M. L., Depp, C. A., & Granholm, E. (2021). Real world sedentary behavior and activity levels in patients with schizophrenia and controls: An ecological momentary assessment study. Mental health and physical activity, 20, 100364.

9. Freeman, D., Taylor, K. M., Molodynski, A., & Waite, F. (2019). Treatable clinical intervention targets for patients with schizophrenia. Schizophrenia Research, 211, 44-50.

10. Fennell, M. J. (1998). Cognitive therapy in the treatment of low self-esteem. Advances in Psychiatric Treatment, 4(5), 296-304.

11. Seligman, M. E. P., Steen, T. A., Park, N., & Peterson, C. (2005). Positive psychology progress: Empirical validation of interventions. American Psychologist, 60, 410–421.

12. Freeman, D., & Freeman, J. (2012). You can be happy: the scientifically proven way to change how you feel. Pearson UK.

13. Freeman, D., Pugh, K., Dunn, G., Evans, N., Sheaves, B., Waite, F., Černis, E., Lister, R., & Fowler, D. (2014). An early Phase II randomised controlled trial testing the effect on persecutory delusions of using CBT to reduce negative cognitions about the self: the potential benefits of enhancing self confidence. Schizophrenia research, 160, 186-192.

For peer review only

14. Morina, N., Ijntema, H., Meyerbröker, K., & Emmelkamp, P. (2015). Can virtual reality exposure therapy gains be generalised to real-life? A meta-analysis of studies applying behavioural assessments. Behaviour Research and Therapy, 74, 18-24.

15. Gujjar, K. R., van Wijk, A., Kumar, R., & de Jongh, A. (2019). Efficacy of virtual reality exposure therapy for the treatment of dental phobia in adults: A randomized controlled trial. Journal of anxiety disorders, 62, 100-108.

16. Bentz, D., Wang, N., Ibach, M. K., Schicktanz, N. S., Zimmer, A., Papassotiropoulos, A., & de Quervain, D. J. (2021). Effectiveness of a stand-alone, smartphone-based virtual reality exposure app to reduce fear of heights in real-life: a randomized trial. NPJ digital medicine, 4(1), 16.

17. Freeman, D., Haselton, P., Freeman, J., Spanlang, B., Kishore, S., Albery, E., Denne, M., Brown, P., Slater, M., & Nickless, A. (2018). Automated psychological therapy using immersive virtual reality for the treatment of the fear of heights: a single-blind parallel-group randomised controlled trial. Lancet Psychiatry, 5, 625-632.

18. Freeman, D., Freeman, J., Ahmed, M., Haynes, P., Beckwith, H., Rovira, A., Miguel, A.L., Ward, R., Bousfield, M., Riffiod, L., Kabir, T., Waite, F., & Rosebrock, L. (in revision). Automated VR therapy for improving positive self-beliefs and psychological wellbeing in young patients with psychosis: a proof of concept evaluation of Phoenix VR self-confidence therapy. Behavioural and Cognitive Psychotherapy.

19. Freeman, D., Rosebrock, L., Loe, B.S., Saidel, S., Freeman, J., & Waite, F. (2023). The Oxford Positive Self Scale: psychometric development of an assessment of cognitions associated with psychological well-being. Psychological Medicine, pp.1-9.

20. Allan, S., Gilbert, P., (1995). Social comparison scale: psychometric properties and relationship to psychopathology. Personality and Individual Differences, 19, 293-299.

21. Tennant, R., Hiller, L., Fishwick, R., Platt, S., Joseph, S., Weich, S. et al. (2007). The Warwick-Edinburgh Mental Well-being Scale (WEMWBS): development and UK validation. Health and Quality of Life Outcomes, 5, 63-80.

22. Waite, F. et al. Everyday Confidence Scale. Unpublished.

23. Kroenke, K., Spitzer, R., & Williams, J. (2001). The PHQ-9. Journal of General Internal Medicine, 16, 606-613.

24. Beck, A. T., & Steer, R. A. (1988). Manual for the Beck Hopelessness Scale. San Antonio, TX: Psychological Corp.

25. Gard, D. E., Gard, M. G., Kring, A. M., & John, O. P. (2006). Anticipatory and consummatory components of the experience of pleasure: A scale development study. Journal of Research in Personality, 40(6), 1086-1102.

26. Spitzer, R. L., Kroenke, K., Williams, J. B. W., & Löwe, B. (2006). A brief measure for assessing generalized anxiety disorder: The GAD-7. Archives of Internal Medicine, 166, 1092-1097.

For peer review only

27. Freeman, D., Loe, B.S., Kingdon, D., Startup, H., Molodynski, A., Rosebrock, L., Brown, P., Sheaves, B., Waite, F., & Bird, J.C. (2019). The revised Green et al., Paranoid Thoughts Scale (R-GPTS): psychometric properties, severity ranges, and clinical cut-offs. Psychological Medicine, 51, 244-253.

28. Law, H., Neil, S., Dunn, G., & Morrison, A. (2014). Psychometric properties of the Questionnaire about the Process of Recovery (QPR). Schizophrenia Research, 156, 184-189.

29. Jolley, S., Garety, P. A., Ellett, L., Kuipers, E., Freeman, D., Bebbington, P. E., Fowler, D. G., & Dunn, G. (2006). A validation of a new measure of activity in psychosis. Schizophrenia Research, 85(1–3), 288–295.

30. Herdman, M., Gudex, C., Lloyd, A., Janssen, M., Kind, P., Parkin, D., Bonsel, G., & Badia, X. (2011). Development and preliminary testing of the new five-level version of EQ-5D (EQ-5D-5L). Quality of Life Research, 20, 1727-1736.

31. Keetharuth, A., Brazier, J., Connell, J., Bjorner, J., Carlton, J., Taylor Buck, E., Ricketts, T., McKendrick, K., Browne, J., Croudace, T., & Barkham, M. (2018). Recovering Quality of Life (ReQoL): a new generic self-reported outcome measure for use with people experiencing mental health difficulties. British Journal of Psychiatry, 212, 42-49.

32. Beecham, J. & Knapp, M. (1992). Costing psychiatric interventions. In Measuring Mental Health Needs (Eds. G Thornicroft, CR Brewin, JK Wing). pp 163-184. London: Gaskell.

33. Attkisson, C.C. & Greenfield, T.K. (1999). The UCSF Client Satisfaction Scales: I. The Client Satisfaction Questionnaire-8. Pp 1333-1346. In The use of psychological testing for treatment, planning and outcome assessments (Ed. M.E. Maruish). Second edition. Mahwah, NJ: Lawrence Erlbaum Associates.

34. Attkisson, C.C. & Zwick, R. (1982). The client satisfaction questionnaire. Evaluation and Program Planning, 5, 233–237.

35. Freeman, D., Rosebrock, L., Waite, F., Loe, B.S., Kabir, T., Petit, A., Dudley, R., Chapman, K., Morrison, A., O'Regan, E., Aynsworth, C., Jones, J., Murphy, E., Powling, R., Peel, H., Walker, H., Bryne, R., Freeman, J., Rovira, A., Galal, U., Yu, L-M., Clark, D.M., & Lambe. S. (2022). Virtual reality (VR) therapy for patients with psychosis: satisfaction and side effects. Psychological Medicine, 53, 4373–4384.

36. Schulz, K. F., Altman, D. G., & Moher, D. (2010). CONSORT 2010 Statement: updated guidelines for reporting parallel group randomised trials. BMC Medicine, 8(1), 18. ttps://doi.org/10.1186/1741-7015-8-18

For peer review only

Table 1. Summary of objectives and assessment measures

	Objectives	Outcome Measures
Primary	Test whether the virtual reality treatment leads to improvement in positive self-beliefs.	Oxford Positive Self Scale (OxPos)
Secondary	Test whether the VR therapy improves social comparison and psychological well-being.	Social Comparison Scale, Warwick-Edinburgh Wellbeing Scale
	Test whether the VR therapy leads to reductions in depression, negative self-beliefs, everyday self-confidence, hopelessness, anhedonia, anxiety, and paranoia and increases in perceptions of recovery, meaningful activity, and quality of life.	Patient Health Questionnaire -9, Brief Core Schema Scale, Everyday Confidence Scale, Beck Hopelessness Scale, Temporal Experience of Pleasure Scale, GAD-7, Revised Green et al Paranoid Thoughts Scale, Questionnaire of Process of Recovery, time budget, EQ-5D-5L, ReQol.
	Assess patient satisfaction with the VR therapy.	Modified version of the Client Satisfaction Questionnaire.
	Assess side effects of the VR therapy	Oxford – VR Side Effects Scale

For peer review only

Figure Legends

Figure 1. Trial flow diagram

For peer review only

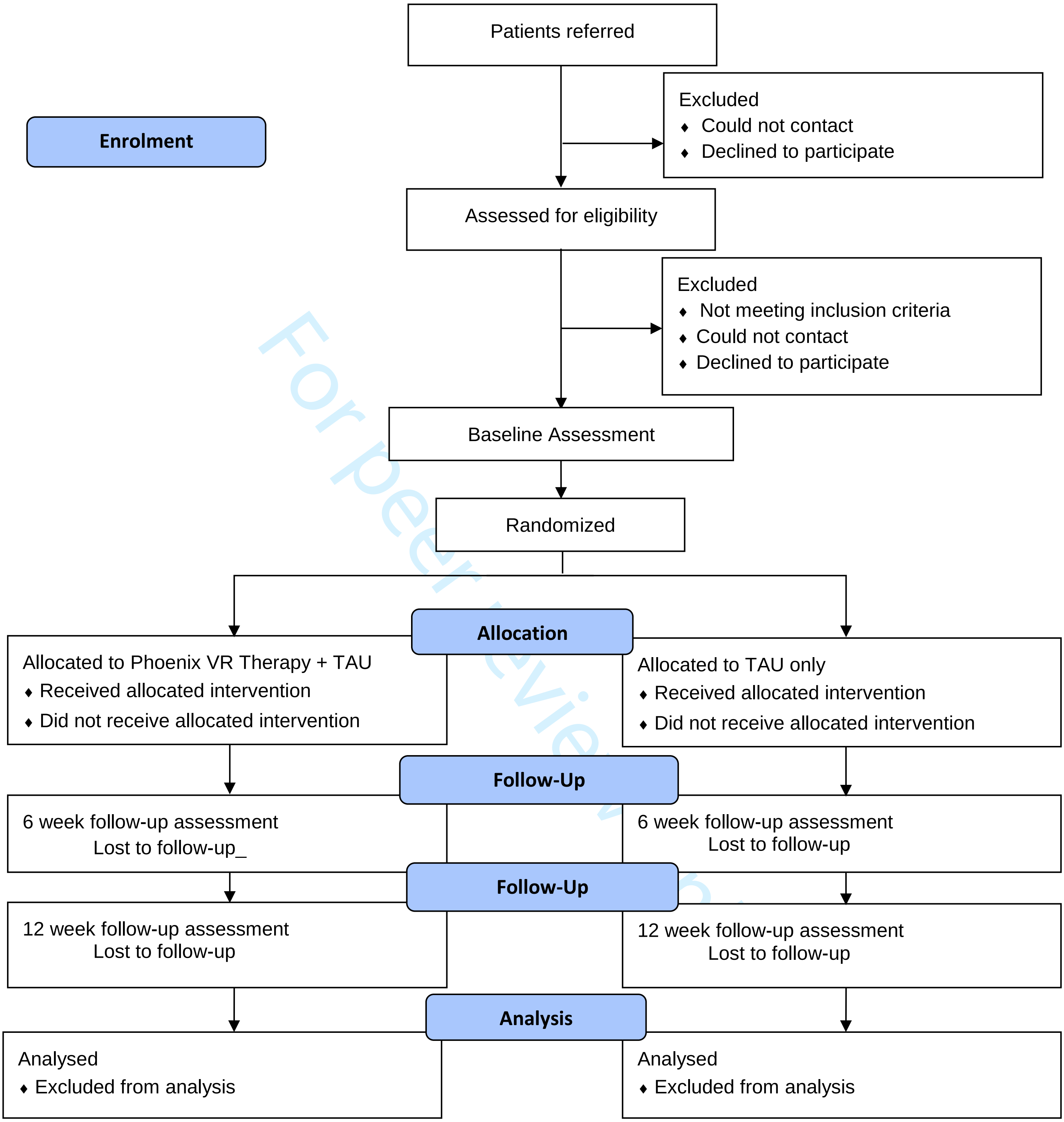
Patients referred
Excluded
Could not contact
Declined to participate
Enrolment
Assessed for eligibility
Excluded
Not meeting inclusion criteria
Could not contact
Declined to participate
Baseline Assessment
Randomized
Allocation
Allocated to Phoenix VR Therapy + TAU
Received allocated intervention
Did not receive allocated intervention
Allocated to TAU only
Received allocated intervention
Did not receive allocated intervention
Follow-Up
6 week follow-up assessment
Lost to follow-up_
6 week follow-up assessment
Lost to follow-up
Follow-Up
12 week follow-up assessment
Lost to follow-up
12 week follow-up assessment
Lost to follow-up
Analysis
Analysed
Excluded from analysis
Analysed
Excluded from analysis

SPIRIT
STANDARD PROTOCOL ITEMS: RECOMMENDATIONS FOR INTERVENTIONAL TRIALS

SPIRIT 2013 Checklist: Recommended items to address in a clinical trial protocol and related documents*

Section/item	Item No	Description	Page
**Administrative information**			
Title	1	Descriptive title identifying the study design, population, interventions, and, if applicable, trial acronym	1
Trial registration	2a	Trial identifier and registry name. If not yet registered, name of intended registry	6
	2b	All items from the World Health Organization Trial Registration Data Set	
Protocol version	3	Date and version identifier	6
Funding	4	Sources and types of financial, material, and other support	13
Roles and responsibilities	5a	Names, affiliations, and roles of protocol contributors	1
	5b	Name and contact information for the trial sponsor	6
	5c	Role of study sponsor and funders, if any, in study design; collection, management, analysis, and interpretation of data; writing of the report; and the decision to submit the report for publication, including whether they will have ultimate authority over any of these activities	
	5d	Composition, roles, and responsibilities of the coordinating centre, steering committee, endpoint adjudication committee, data management team, and other individuals or groups overseeing the trial, if applicable (see Item 21a for data monitoring committee)	
**Introduction**			
Background and rationale	6a	Description of research question and justification for undertaking the trial, including summary of relevant studies (published and unpublished) examining benefits and harms for each intervention	5
	6b	Explanation for choice of comparators	5
Objectives	7	Specific objectives or hypotheses	5
Trial design	8	Description of trial design including type of trial (eg, parallel group, crossover, factorial, single group), allocation ratio, and framework (eg, superiority, equivalence, noninferiority, exploratory)	6

For peer review only

Methods: Participants, interventions, and outcomes

Study setting	9	Description of study settings (eg, community clinic, academic hospital) and list of countries where data will be collected. Reference to where list of study sites can be obtained	7
Eligibility criteria	10	Inclusion and exclusion criteria for participants. If applicable, eligibility criteria for study centres and individuals who will perform the interventions (eg, surgeons, psychotherapists)	7
Interventions	11a	Interventions for each group with sufficient detail to allow replication, including how and when they will be administered	9
	11b	Criteria for discontinuing or modifying allocated interventions for a given trial participant (eg, drug dose change in response to harms, participant request, or improving/worsening disease)	
	11c	Strategies to improve adherence to intervention protocols, and any procedures for monitoring adherence (eg, drug tablet return, laboratory tests)	
	11d	Relevant concomitant care and interventions that are permitted or prohibited during the trial	10
Outcomes	12	Primary, secondary, and other outcomes, including the specific measurement variable (eg, systolic blood pressure), analysis metric (eg, change from baseline, final value, time to event), method of aggregation (eg, median, proportion), and time point for each outcome. Explanation of the clinical relevance of chosen efficacy and harm outcomes is strongly recommended	8
Participant timeline	13	Time schedule of enrolment, interventions (including any run-ins and washouts), assessments, and visits for participants. A schematic diagram is highly recommended (see Figure)	17
Sample size	14	Estimated number of participants needed to achieve study objectives and how it was determined, including clinical and statistical assumptions supporting any sample size calculations	12
Recruitment	15	Strategies for achieving adequate participant enrolment to reach target sample size	7

Methods: Assignment of interventions (for controlled trials)

Allocation:

Sequence generation	16a	Method of generating the allocation sequence (eg, computer-generated random numbers), and list of any factors for stratification. To reduce predictability of a random sequence, details of any planned restriction (eg, blocking) should be provided in a separate document that is unavailable to those who enrol participants or assign interventions	6

For peer review only - http://bmjopen.bmj.com/site/about/guidelines.xhtml

Allocation concealment mechanism	16b	Mechanism of implementing the allocation sequence (eg, central telephone; sequentially numbered, opaque, sealed envelopes), describing any steps to conceal the sequence until interventions are assigned	6
Implementation	16c	Who will generate the allocation sequence, who will enrol participants, and who will assign participants to interventions	6
Blinding (masking)	17a	Who will be blinded after assignment to interventions (eg, trial participants, care providers, outcome assessors, data analysts), and how	6
	17b	If blinded, circumstances under which unblinding is permissible, and procedure for revealing a participant's allocated intervention during the trial	
**Methods: Data collection, management, and analysis**			
Data collection methods	18a	Plans for assessment and collection of outcome, baseline, and other trial data, including any related processes to promote data quality (eg, duplicate measurements, training of assessors) and a description of study instruments (eg, questionnaires, laboratory tests) along with their reliability and validity, if known. Reference to where data collection forms can be found, if not in the protocol	8
	18b	Plans to promote participant retention and complete follow-up, including list of any outcome data to be collected for participants who discontinue or deviate from intervention protocols	
Data management	19	Plans for data entry, coding, security, and storage, including any related processes to promote data quality (eg, double data entry; range checks for data values). Reference to where details of data management procedures can be found, if not in the protocol	Ethics protocol
Statistical methods	20a	Statistical methods for analysing primary and secondary outcomes. Reference to where other details of the statistical analysis plan can be found, if not in the protocol	12
	20b	Methods for any additional analyses (eg, subgroup and adjusted analyses)	12
	20c	Definition of analysis population relating to protocol non-adherence (eg, as randomised analysis), and any statistical methods to handle missing data (eg, multiple imputation)	11
**Methods: Monitoring**			
Data monitoring	21a	Composition of data monitoring committee (DMC); summary of its role and reporting structure; statement of whether it is independent from the sponsor and competing interests; and reference to where further details about its charter can be found, if not in the protocol. Alternatively, an explanation of why a DMC is not needed	6

	21b	Description of any interim analyses and stopping guidelines, including who will have access to these interim results and make the final decision to terminate the trial	
Harms	22	Plans for collecting, assessing, reporting, and managing solicited and spontaneously reported adverse events and other unintended effects of trial interventions or trial conduct	10
Auditing	23	Frequency and procedures for auditing trial conduct, if any, and whether the process will be independent from investigators and the sponsor	
**Ethics and dissemination**			
Research ethics approval	24	Plans for seeking research ethics committee/institutional review board (REC/IRB) approval	12
Protocol amendments	25	Plans for communicating important protocol modifications (eg, changes to eligibility criteria, outcomes, analyses) to relevant parties (eg, investigators, REC/IRBs, trial participants, trial registries, journals, regulators)	
Consent or assent	26a	Who will obtain informed consent or assent from potential trial participants or authorised surrogates, and how (see Item 32)	
	26b	Additional consent provisions for collection and use of participant data and biological specimens in ancillary studies, if applicable	
Confidentiality	27	How personal information about potential and enrolled participants will be collected, shared, and maintained in order to protect confidentiality before, during, and after the trial	Ethics protocol
Declaration of interests	28	Financial and other competing interests for principal investigators for the overall trial and each study site	13
Access to data	29	Statement of who will have access to the final trial dataset, and disclosure of contractual agreements that limit such access for investigators	Ethics protocol
Ancillary and post-trial care	30	Provisions, if any, for ancillary and post-trial care, and for compensation to those who suffer harm from trial participation	
Dissemination policy	31a	Plans for investigators and sponsor to communicate trial results to participants, healthcare professionals, the public, and other relevant groups (eg, via publication, reporting in results databases, or other data sharing arrangements), including any publication restrictions	
	31b	Authorship eligibility guidelines and any intended use of professional writers	
	31c	Plans, if any, for granting public access to the full protocol, participant-level dataset, and statistical code	13

For peer review only - http://bmjopen.bmj.com/site/about/guidelines.xhtml

Appendices

Informed consent materials	32	Model consent form and other related documentation given to participants and authorised surrogates
Biological specimens	33	Plans for collection, laboratory evaluation, and storage of biological specimens for genetic or molecular analysis in the current trial and for future use in ancillary studies, if applicable

*It is strongly recommended that this checklist be read in conjunction with the SPIRIT 2013 Explanation & Elaboration for important clarification on the items. Amendments to the protocol should be tracked and dated. The SPIRIT checklist is copyrighted by the SPIRIT Group under the Creative Commons "Attribution-NonCommercial-NoDerivs 3.0 Unported" license.

For peer review only
